# Application of Artificial Intelligence in the MRI Classification Task of Human Brain Neurological and Psychiatric Diseases: A Scoping Review

**DOI:** 10.3390/diagnostics11081402

**Published:** 2021-08-03

**Authors:** Zhao Zhang, Guangfei Li, Yong Xu, Xiaoying Tang

**Affiliations:** 1 715-3 Teaching Building No.5, Department of Biomedical Engineering, School of Life Sciences, Beijing Institute of Technology, 5 South Zhongguancun Road, Haidian District, Beijing 100081, China; 3120195716@bit.edu.cn (Z.Z.); 3120185691@bit.edu.cn (G.L.); 2 Department of Cardiology, Chinese PLA General Hospital, Beijing 100853, China; xuyong@plagh.cn

**Keywords:** artificial intelligence, machine learning, deep learning, human brain-related diseases, magnetic resonance image

## Abstract

Artificial intelligence (AI) for medical imaging is a technology with great potential. An in-depth understanding of the principles and applications of magnetic resonance imaging (MRI), machine learning (ML), and deep learning (DL) is fundamental for developing AI-based algorithms that can meet the requirements of clinical diagnosis and have excellent quality and efficiency. Moreover, a more comprehensive understanding of applications and opportunities would help to implement AI-based methods in an ethical and sustainable manner. This review first summarizes recent research advances in ML and DL techniques for classifying human brain magnetic resonance images. Then, the application of ML and DL methods to six typical neurological and psychiatric diseases is summarized, including Alzheimer’s disease (AD), Parkinson’s disease (PD), major depressive disorder (MDD), schizophrenia (SCZ), attention-deficit/hyperactivity disorder (ADHD), and autism spectrum disorder (ASD). Finally, the limitations of the existing research are discussed, and possible future research directions are proposed.

## 1. Introduction

Magnetic resonance imaging (MRI), as a non-invasive medical imaging technique, has been widely used in the early detection, diagnosis, and treatment of diseases [1]. In the study of the human brain, MRI can not only provide information about the anatomical structure of the brain, but also provides comprehensive multi-parameter information about the function and metabolism [2]. Structural magnetic resonance imaging (sMRI) and functional magnetic resonance imaging (fMRI) have respectively made great progress in the study of human brain structure and function, due to their high spatial resolution [3].

**Figure 1:**
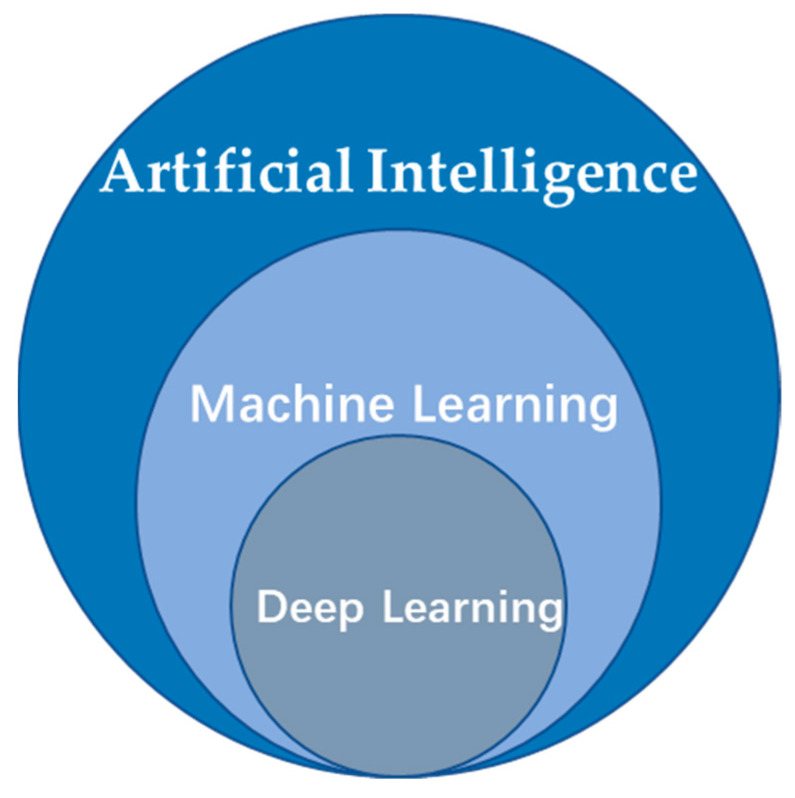
The relationship between AI, ML, and DL.

AI (artificial intelligence) in MRI is a technology with great potential. Based on the principles and application of ML (machine learning), DL (deep learning) is fundamental for developing AI-based algorithms that can achieve improved results, quality, and efficiency [4]. The relationship between AI, ML, and DL is shown in Figure 1.

Machine learning [5,6], as a pattern recognition technology, has already been applied to the medical imaging field. ML usually starts by selecting features that are considered important for making predictions or diagnoses. The ML algorithm then identifies the best combination of these selected features for classifying or computing some metrics for the given image. With the development of AI technology, ML will continue to have a great influence in the future [7].

Deep learning [8] is an end-to-end algorithm featuring automatic feature learning. Since it does not rely on the artificial extraction of features, as in traditional ML, more original features of the data can be obtained [9]. Recently, DL has renewed the potential of neural networks and has been widely applied to natural language processing, image recognition, and other fields, using its powerful feature modeling and learning capabilities. Due to the complexity and unpredictability of the human body and diseases, biological signals and information are detected and expressed according to their manifestations and changing patterns (self-changes and changes after medical intervention). The analysis of the acquired data and information, decision-making, and many other aspects has complicated nonlinear relations, which is suitable for application of neural networks. Furthermore, there exist a number of popular reviews: Noor et al. [10] compared performances of the existing deep learning (DL)-based methods for detecting neurological disorders from MRI data acquired using different modalities, including functional and structural MRI, they also summarized the application of DL and reinforcement learning (RL) in biological data [11,12].

In the diagnosis of human brain-related diseases, such as AD (Alzheimer′s disease) [13], PD (Parkinson’s Disease) [14], MDD (major depressive disorder) [15], SCZ (schizophrenia) [16], ADHD (attention-deficit/hyperactivity disorder) [17], ASD (autism spectrum disorder) [17,18], etc., the use of AI methods to diagnose diseases has achieved satisfactory results [19]. In the radiological research of the human brain, the raw data is mainly the collected magnetic resonance images of the human brain. The main imaging methods include T1-weighted imaging, T2-weighted imaging, diffusion tensor imaging (DTI), diffusion-weighted imaging (DWI), and blood oxygen level-dependent functional magnetic resonance imaging (BOLD-fMRI), etc. After the raw data is preprocessed, images are classified with different ML and DL models, which can overcome the subjective limitations of traditional diagnosis by doctors and realize a transformation from subjective qualitative analysis to objective quantitative analysis.

Differently from traditional two-dimensional images, magnetic resonance images are three-dimensional, or even four-dimensional, spatial images. Considering this feature, we have summarized related ML and DL methods and their applications in human brain-related neurological and psychiatric diseases.

A certain amount of literature works have reviewed the application of ML and DL methods on MRI data [4,20], and some representative examples are listed in Table 1. Some of them reviewed the MRI data of different parts of the human body, including the brain, chest, breast, and others [21]. Others reviewed only ML, while some reviewed only DL methods. Almost no papers simultaneously reviewed ML and DL in human brain neurological and psychiatric diseases in the classification task. As the two most popular research directions in the field of AI in the past decade, it is necessary to conduct a comprehensive review and pay closer attention to human neurological and psychiatric diseases.

This paper’s contribution lies in its systematic and comprehensive introduction to the application of ML and DL methods to human brain magnetic resonance data in neurological and psychiatric diseases. We briefly introduce the principle of the algorithms and then review the related literature on the application of these algorithms. Therefore, the structure of this review can roughly be divided into five parts. In Section 2, the method for this review is introduced. In Section 3 and Section 4, we introduce some popular ML/DL models. In Section 5, we provide a detailed overview of recent studies using AI-based techniques for six human brain-related diseases, and finally, the article is concluded in Section 6.

## 2. Methods

The method described below was guided by scoping review methodological frameworks [28]. The objectives and inclusion and exclusion criteria for this scoping review were prespecified and published in a protocol using the open science framework [29,30].

### 2.1. Search Strategy and Literature Sources

For this review, classification task AI-based methods were searched in databases including PubMed and Web of Science, from January 2011 to May 2021. The search string used in this study was (“Alzheimer’s Disease” or “Parkinson’s Disease” or “Major Depressive Disorder” or “Schizophrenia” or “Attention-Deficit/Hyperactivity Disorder” or “Autism Spectrum Disorder” or “AD” or “PD” or “MDD” or “SCZ” or “ADHD” or “ASD”) and (“machine learning” or “deep learning”) and (“MRI”).

### 2.2. Inclusion Criteria

Articles published between January 2011 and May 2021 on the six specified diseases and written in English, using brain MR images and machine learning or deep learning methods were included. According to effective practice and organization of care review criteria, the following study designs were considered for inclusion: randomized control trials, non-randomized control trials, and controlled before–after studies. Relatively new research was included in this review.

### 2.3. Exclusion Criteria

Articles not written in English, reported before January 2011, not a classification task, not using brain MR images, not the specified diseases, case reports/case series, letters to the editor, opinions, commentaries, conference abstracts, dissertations, and theses were excluded from this review.

### 2.4. Results

**Figure 2:**
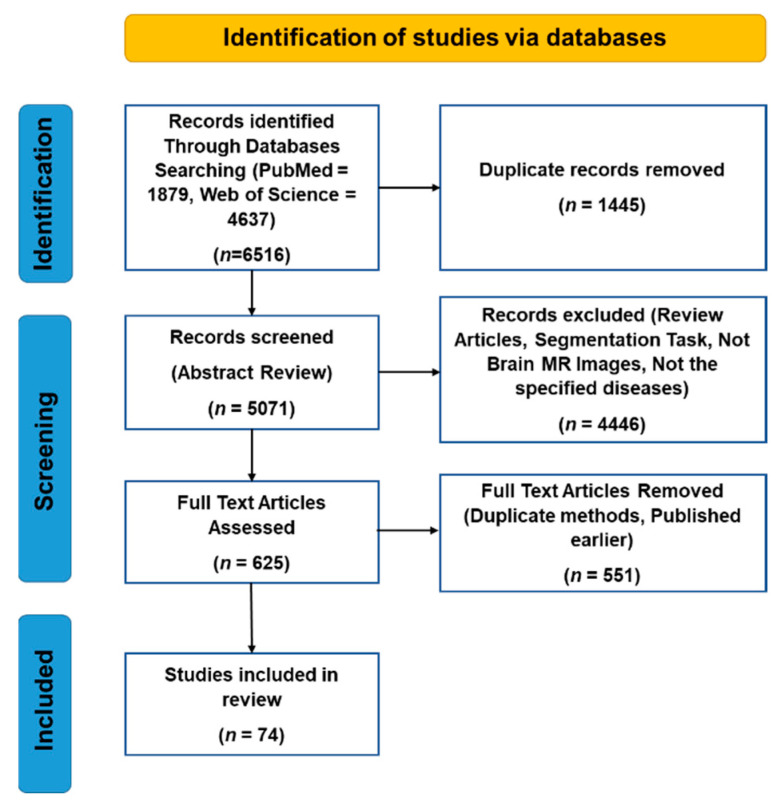
Prisma diagram of the selection process of the research articles of this review.

Initially, 6516 papers were found. After removing duplicates and reviewing the abstracts of these papers, 625 papers were selected for full-text review. This study includes both journal and conference articles. After reviewing the full-text of these papers, 551 papers were excluded, as they used duplicate methods or were published earlier. Finally, 74 papers were studied in this research. Figure 2 illustrates the selection procedure of the articles for this study using a Prisma diagram.

## 3. Related Machine Learning Methods

**Figure 3:**
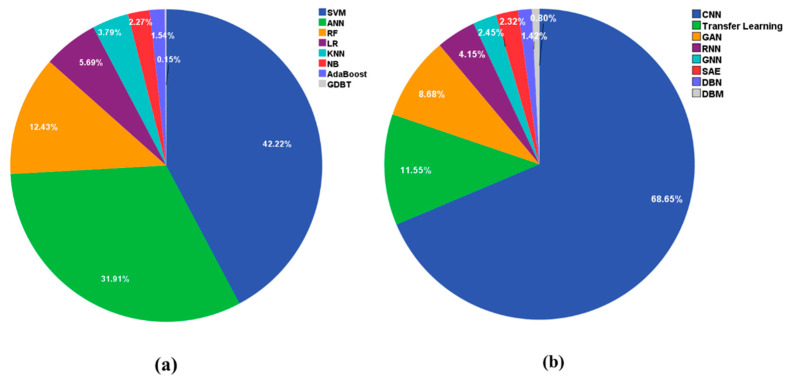
Application of different AI-based models to MRI data. (**a**) The related machine learning methods. Legend, SVM: support vector machine, ANN: artificial neural network, RF: random forests, LR: logistic regression, KNN: K-nearest neighbor, NB: naive Bayes, GDBT: gradient boosting decision tree. (**b**) The related deep learning methods. Legend, CNN: convolutional neural network, GAN: generative adversarial network, RNN: recurrent neural network, GNN: graph neural network, SAE: stacked auto-encoders, DBN: deep belief network, DBM: deep Boltzmann machine.

In the past ten years, many ML models have been used on a variety of MRI data, and we recorded statistics on the commonly used classification algorithms through Web of Science. The results showed that SVM was used in 1973 papers, ANN in 1491 papers, RF in 581 papers, LR in 266 papers, KNN in 177 papers, NB in 106 papers, AdaBoost in 72 papers, and GBDT in 7 papers. Figure 3a lists the most popular ML methods. The first six models are reviewed in this paper.

### 3.1. K-Nearest Neighbor (KNN)

**Figure 4:**
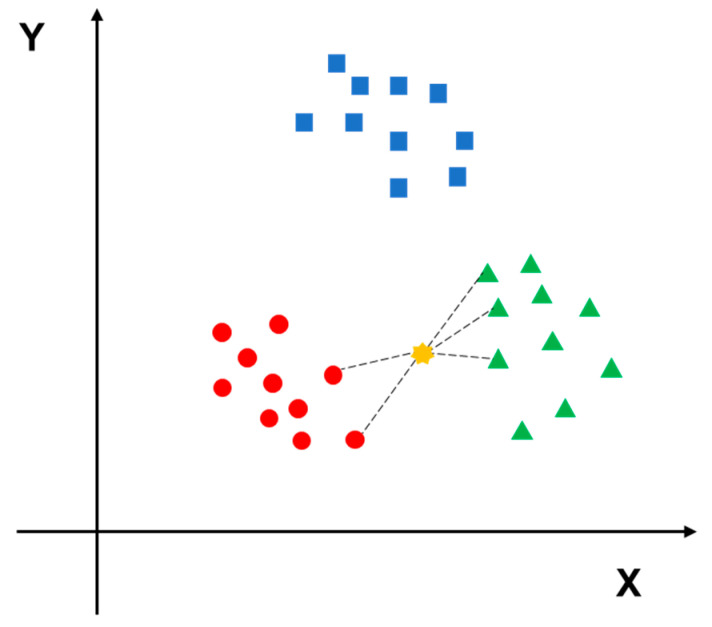
The red circles, blue squares and green triangles represent three different categories of data, the yellow point is the data to be predicted. Since it is closer to the green triangle type, it should be predicted to belong to the green triangle type.

KNN is a comprehensive model that can be used for both classification and regression tasks [31]. The KNN algorithm can be easily understood; if a sample belongs to category A in most of the K closest samples in the sample space, then the sample also belongs to A. When making a classification decision, the method is based only on the type of samples with the largest number among the closest K samples. In other words, the classification decision of the KNN method is only related to a very small number of adjacent samples. A Euclidean function is used to calculate the neighboring distance, which stands for the correlation between two samples [32]. The principle of KNN algorithm is shown in Figure 4.

### 3.2. Naive Bayes (NB)

NB is a simplified model based on the Bayes algorithm, which assumes that attributes are conditionally independent of each other; i.e., no attribute has a larger or smaller weight in affecting the decision result. It calculates the result of individual groups, which are not associated with other variables [33]. NB is a simple and efficient classification model and has been widely used for classification and multi-class predictions [34].

### 3.3. Support Vector Machine (SVM)

**Figure 5:**
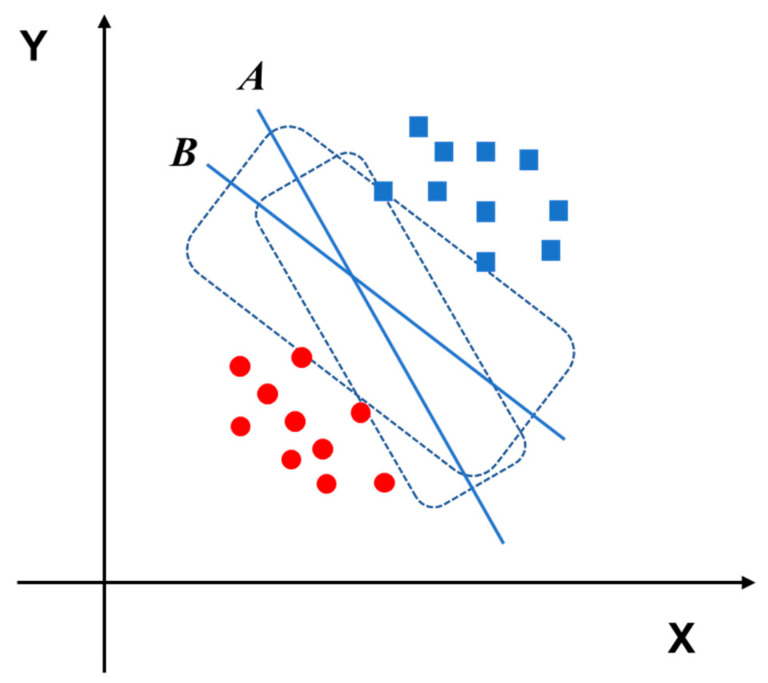
The red circles and blue squares represent two different categories of data. Hyperplane B can classify the red circles and blue squares better than hyperplane A.

As one of the most used ML models, SVM is an algorithm developed for classification tasks. In the SVM model, the features are mapped onto high-dimensional space, and classification is performed to decide the optimal hyperplane [35]. The fundamental concept of the SVM model is to transform the classification problem into a convex quadratic programming problem, which can be solved by the relevant ideas of operations research. Specifically, SVM uses labeled data to generate the best hyperplane through the training step, and the hyperplane can optimally separate the data into different types. This hyperplane is a line of binary classification, and tuning parameters can help to improve the performance of the model [36,37]. A schematic diagram of a hyperplane is illustrated in Figure 5.

### 3.4. Random Forests (RF)

**Figure 6:**
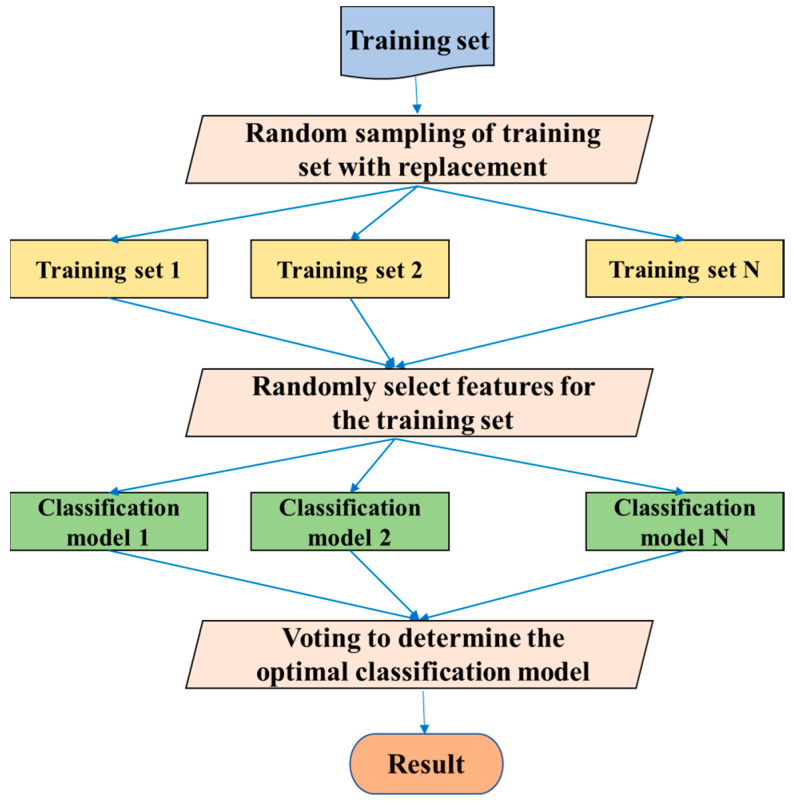
The algorithm flow of RF.

RF was introduced by Leo Breiman [38] and is an ensemble algorithm that is an extension of the bagging idea [39]. Multiple weak classifiers are combined to form RF, and the final result is obtained by voting or averaging these weak classifiers, so that the RF can achieve a higher accuracy and generalization performance. The “random” function makes the model resistant to overfitting, and the “forests” make the result more accurate. RF can be used for either a classification problem or a regression problem. Similarly, the predictor variables can be either categorical or continuous. The algorithm flow of RF is illustrated in Figure 6.

### 3.5. Logistic Regression (LR)

**Figure 7:**
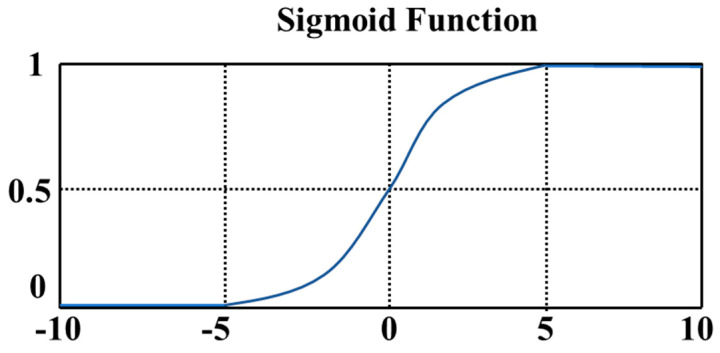
The sigmoid function maps a real number to the interval of (0,1) and can be used for binary classification.

LR was first used by Cornfield et al. [40]. The use of LR has continued to increase in the past thirty years. It is one of the most widely used methods in health science research, especially epidemiological research [41]. The difference between LR and linear regression is that LR assumes that the dependent variable *y* follows a Bernoulli distribution, while linear regression assumes that the dependent variable *y* follows a Gaussian distribution. LR is theoretically supported by linear regression, but LR introduces nonlinear factors through the use of a sigmoid function. A graphical representation of the sigmoid function is shown in Figure 7.

### 3.6. Artificial Neural Network (ANN)

**Figure 8:**
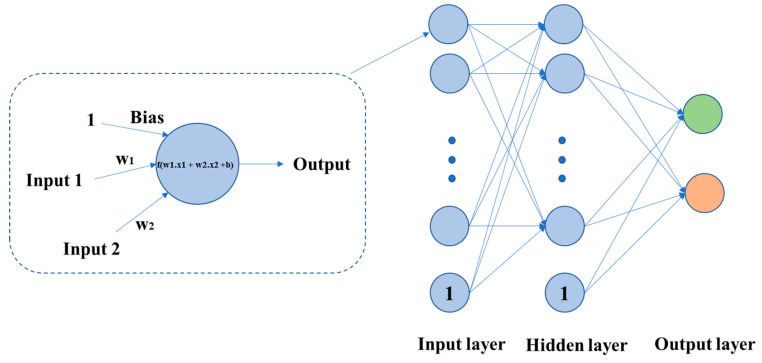
Each neuron is a computing unit, which can be represented by a calculation function f. An ANN is composed of one input layer, several hidden layers, and one output layer.

ANN is a significant model in the field of ML. It is inspired by the working principle of biological nerve cells and combines multiple hierarchical relationships. The neural network structure is formed by interconnected artificial neurons. The signal transmission between these neurons can be simulated by a mathematical expression, so that a nonlinear relationship between an input and output can be established and visualized. ANN can simulate any nonlinear functions with different structures, and therefore it can be used to process nonlinear systems or black-box models with more complex internal expressions. The schematic diagrams of a neuron and an ANN are shown in Figure 8.

## 4. Related Deep Learning Methods

DL models have also been widely used on MRI data. Figure 3b lists the most popular DL methods. The first six models are reviewed in this paper. The statistics results showed that CNN was used in 1986 papers, transfer learning in 334 papers, GAN in 251 papers, RNN in 120 papers, GNN in 71 papers, SAE in 67 papers, DBN in 41 papers, and DBM in 23 papers.

### 4.1. Stacked Auto-Encoders (SAE)

**Figure 9:**
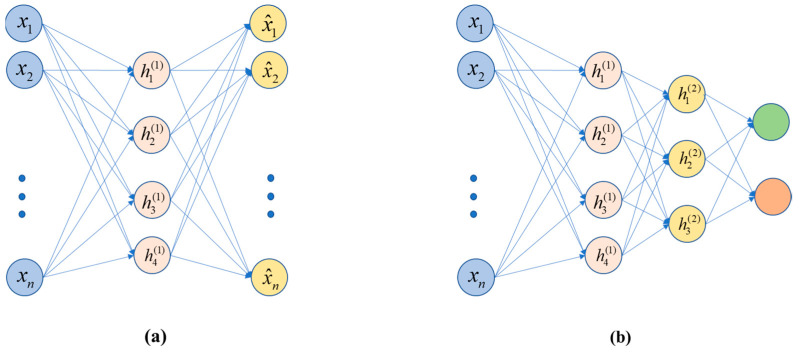
(**a**) An AE, including encoding and decoding, and (**b**) an SAE without the decoding process.

The so-called auto-encoder (AE) is an unsupervised learning process, i.e., there is no need for a label. The self-encoding is accomplished by making the label of each sample *y* equal to *x*, which is the data *x* of each sample, and the label is also *x*. Self-encoding generates the label itself, and the label is the sample data itself. The optimization goal during training is to make the output value as close as possible to the input value, preferably the same. This can be understood in this way: the input data represents some information, which is initially represented by the data of a certain dimension; then, after being encoded by the intermediate hidden layer, the dimension is compressed (reduced), and finally it is restored to data that is very close to the original information. The basic realization of the “stacked” process trains the above AE structure and discards the decoding process. Thus, it can be seen that the code contributes to dimensionality reduction and feature extraction. The code at this time is taken as input, and input into the new AE structure, for training. To date, a variety of stackable autoencoders have been proposed, such as denoising auto-encoders (DAE) [42], sparse auto-encoders (sparse AE) [43], and variational auto-encoders (VAE) [44]. The basic structures of an AE and an SAE are shown in Figure 9.

### 4.2. D Convolutional Neural Network (2D-CNN)

Convolutional neural networks (CNN) were proposed by Yann LeCun and evolved from multi-layer perceptron (MLP) [45]. The greatly reduce the number of parameters through weight sharing, making a neural network easy to optimize and resistant to overfitting. A CNN is a feedforward neural network, and it is specially designed to process two-dimensional data. The structure parameters in the network can be optimized through a backpropagation algorithm with chain rules. CNN is considered the first truly successful robust DL method to be based on a multi-layer hierarchical network.

**Figure 10:**
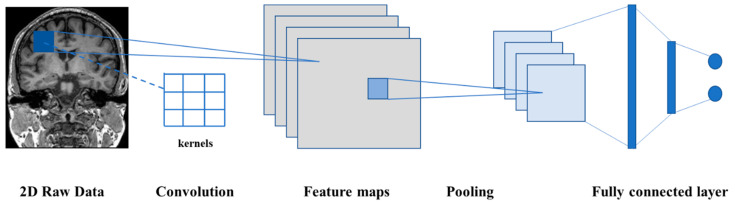
The convolution of a two-dimensional picture calculates the output with convolution by moving the convolution kernel step by step on the picture, down-sampling through pooling layer, and finally connecting the results of the output layer through several fully connected layers.

Developed from artificial neural networks, a CNN can preserve the spatial relationship between data. A CNN model consists of several types of layer, including an input layer, convolutional layer, pooling layer, fully connected layer, and output layer. CNN has multiple layers of convolutions and activations, which makes it able to form a highly efficient representation of the input data [46]. Moreover, the fully connected layers compute the final outputs at the end of the model [24]. The basic architecture of a 2D-CNN is shown in Figure 10. Since MRI images are three-dimensional, the input layer needs to convert the 3D human brain image into one in 2D space, and then input it into the corresponding network model.

### 4.3. D Convolutional Neural Network (3D-CNN)

Compared with 2D convolution, 3D convolution on the entire MRI image can capture potential 3D structural information, which is essential for discrimination [47]. Three-dimensional CNN has shown excellent performances in AD and MCI classification [48] and is a supervised learning framework that learns discriminant features from training data. The design of the network structure is inspired by the functioning of the human eye [47]. In recent years, convolutional neural networks have been widely applied to the fields of visual computing and artificial intelligence. Especially, in the field of visual recognition the use of 3D convolutional neural networks is developed rapidly.

**Figure 11:**
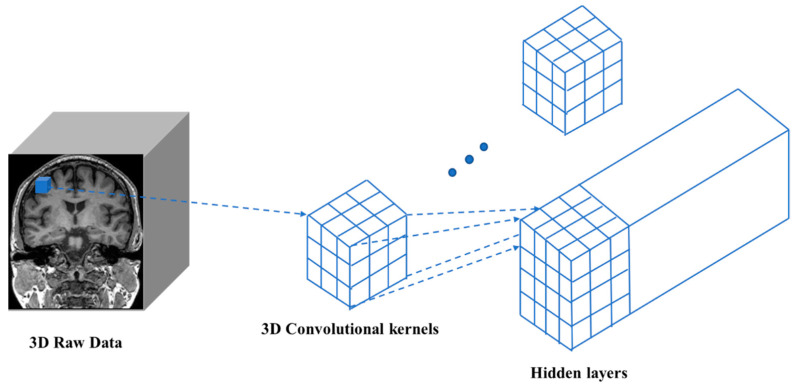
Similar to 2D convolution, the input image, convolution kernel, and final output are all three-dimensional.

Three-dimensional convolution differs from 2D convolution in that the input image has an additional depth dimension. In this case, the input size (channel, depth, height, width) and the convolution kernel have one more dimension. Thus, a sliding window operation is performed on the height and width dimensions, as well as the depth dimension, to obtain a value in the output 3D image. Exploiting 1D and 2D CNNs, a 3D-CNN can simultaneously extract spectral and spatial features from the input data. These features of 3D-CNNs are very useful for analyzing volume data in the field of medical imaging. A schematic diagram of 3D convolution is shown in Figure 11.

### 4.4. Recurrent Neural Network (RNN)

RNNs are obtained by connecting the output of neurons of a feedforward network to their inputs [49]. “Recurrent” means that the network model performs the same operation on each element, and the output of the model depends on previous calculations. The RNN with pattern recognition was introduced by Hopfield in 1982 [50]. As a part of DL techniques, RNN is suitable for processing temporal data, such as fMRI, because it models the temporal correlations among data explicitly with its recurrent structure. Recently, more and more researchers have become interested in using RNN to model fMRI signals [51,52]. Specifically, Güçlü et al. [53] used RNN to predict brain activity response nature activity, to investigate the expression of complex visual and auditory information in the brain.

**Figure 12:**
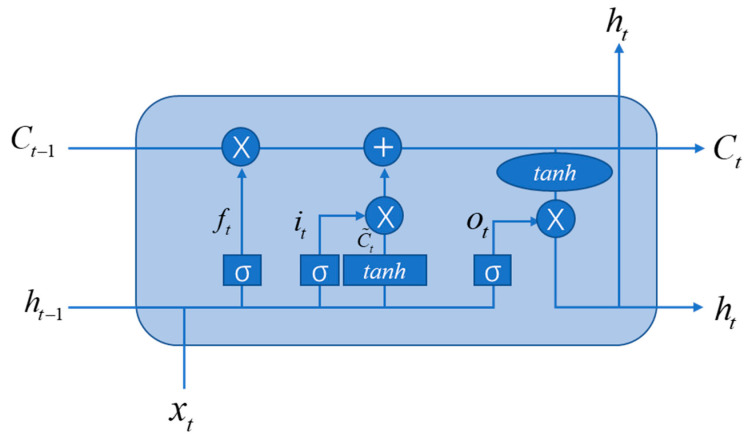
The basic architecture of LSTM.

RNNs can be regarded as deep networks with shared parameters at each layer. This results in the problem of vanishing gradients. To solve this problem, the long-short-term memory (LSTM) architecture was proposed by Hochreiter and Schmidhuber [54]. LSTM is composed of a memory cell *C_t_*, a forget gate *f_t_*, an input gate *i_t_*, and an output gate *o_t_*. The basic architecture of LSTM is shown in Figure 12.

### 4.5. Graph Neural Network (GNN)

The human brain can be regarded as a graph structure, in which different brain areas can be regarded as nodes and the relationship between these areas can be regarded as edges. The graph structure is represented by *G* = {*V*, *E*, *A*}, where *V* represents a set of nodes, and |*V*| = *n* means there are *n* nodes in the graph; *E* represents a set of edges, and *A* is an adjacency matrix, which defines the interconnection between nodes. 

**Figure 13:**
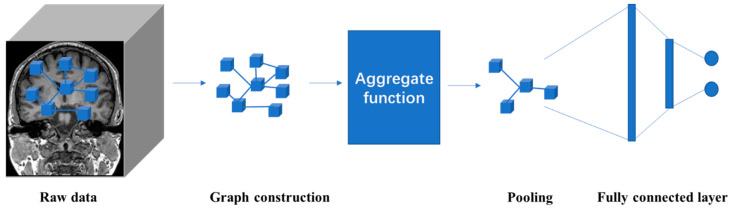
The connections between different brain areas can be abstracted into a graph structure. The high-level representation of the graph structure is learned through aggregation functions, and the output layer is connected through the fully connected layer to obtain the classification result.

In the past few years, inspired by DL models like CNNs, RNNs, and AEs, new generalizations and definitions of important operations have been rapidly proposed to deal with the complexity of graphics data. Sperduti et al. [55] first applied neural networks to directed acyclic graphs, which motivated early studies on GNNs. The concept of graph neural network was originally proposed by Gori et al. and further elaborated by Scarselli et al. and Gallicchio et al. [56,57,58]. The information of the entire graph is represented by an iterative aggregation of neighbor information. This process is time-consuming in computation, and recently efforts have been made to overcome this challenge [59,60]. The representative algorithms for graph neural networks include DeepWalk [61], GCN [62], CTDNE [63], and JODIE [64]. Encouraged by the success of CNNs in the field of computer vision, many methods have been developed to redefine the concept of graph data convolution. These methods are in the category of convolutional graph neural networks (ConvGNNs), which can be divided into spectral-based and spatial-based approaches. The basic architecture of GNN is shown in Figure 13.

### 4.6. Generative Adversarial Network (GAN)

GAN was proposed by Goodfellow et al. [65] and is one of the most effective methods for unsupervised learning of complex distributions of recent years. A GAN contains two key components: the generative model (G) and the discriminative model (D). Neural networks are usually exploited as G and D, but this is not fixed. In other words, G and D are not required to both be neural networks, they can be the functions that can realize the corresponding generation and distinction.

**Figure 14:**
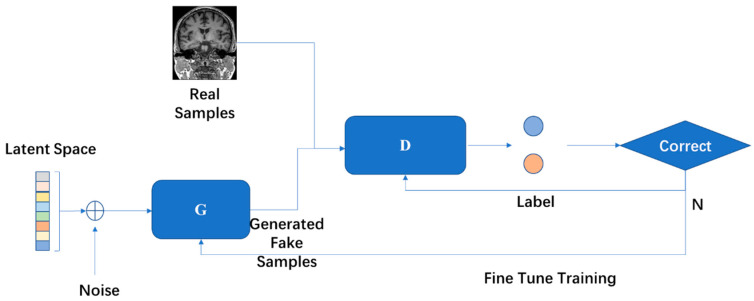
Two models are trained in the framework of GAN at the same time. The training procedure of G is to maximize the error probability of D.

GAN has been extensively explored by researchers, and there have been many conclusive works. Creswell et al. [66] classified GAN models in terms of the network architecture and loss function. Hong et al. [67] summarized the development of the GAN model from the perspective of learning methods, including supervised learning, unsupervised learning, and theoretical analysis. Guo et al. [68] focused on modifying the model structure, developing the theory, and the use of GAN. The expansion of the data set is also an important function of GAN. Different forms of GAN are receiving more and more attention in biomedical research [69]. The basic architecture of GAN is illustrated in Figure 14.

### 4.7. Transfer Learning

Transfer learning is a DL method that transfers knowledge from one domain (source domain) to another domain (target domain), so that better learning results can be achieved in the target domain. Since researchers in brain imaging deal with small datasets, transfer learning might be an approach that improves results [70,71,72].

**Figure 15:**
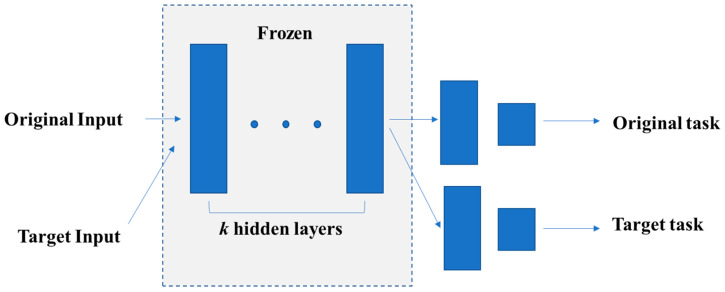
Transfer learning first trains the parameters in the original task. Then, it freezes *k* hidden layers and uses these *k* layers for the target task.

Transfer learning first trains a basic network on a large sample dataset and then migrates the first k layers of the basic network to that of the target network. Based on this, the remaining layers of the target network are randomly initialized and trained on the target data set. During the training process, the parameters of the first *k* layers are usually frozen to avoid overfitting during backpropagation. Specifically, whether to fine-tune the first *k* layers of the target network depends on the size of the target data set and the quantity of the parameters in the first *k* layers. If the target data set is small and the number of parameters is large, fine-tuning may lead to overfitting. Otherwise, if the target data set is large or the number of parameters is small, then overfitting can be avoided, and transfer learning can contribute to performance improvements [73]. This is an efficient approach for improving the training effect on small sample data sets. Since the current MRI data volume is far from sufficient, the application of transfer learning in MRI is necessary. Figure 15 is the schematic diagram of transfer learning.

## 5. Applications in Human Brain MRI Image Classification Tasks

### 5.1. Alzheimer’s Disease 

Alzheimer′s disease is a neurodegenerative disease with a slow onset and worsens over time. This disease accounts for 60% to 70% of the causes of dementia [74]. One of the most common early symptoms of this disease is the loss of short-term memory. As the disease gradually progresses, the patients suffer from the following symptoms: language disorder, disorientation, emotional instability, loss of motivation, inability to take care of themselves, and many behavioral problems [74]. Neuroimaging is an important method for assisting in the diagnosis of AD. Researchers have achieved unparalleled results by analyzing MRI images through AI methods.

ML models usually achieve good performances on small datasets. Battineni et al. [75] used a total of 373 MRI tests to investigate 14 distinct features related to AD diagnosis. Four ML models: NB, ANN, KNN, and SVM were exploited to validate the model performance. The joint model with limited features achieved the best accuracy, of 98%. Dyrba et al. [76] used a multimodal method, with T1, DTI, and resting-state fMRI (rs-fMRI) as inputs to an SVM classifier. DTI obtained the highest accuracy and the multimodal analysis obtained the same accuracy. Moradi et al. [77] created a classifier and used the MRI data along with age and behavioral data as inputs to a random forest. This method obtained an accuracy of 81.72% on progressive mild cognitive impairment (pMCI) and stable mild cognitive impairment (sMCI). Liu et al. [78] exploited multiple weak classifiers, which were constructed following the sparse representation-based classification (SRC) method, and combined the results of these classifiers to produce a final result.

Although the dataset is not large enough, many researchers have tried to use DL models. Odusami et al. [79] used a fine-tuned ResNet18 network to perform seven binary classifications, based on the principle of transfer learning. Yang et al. [80] proposed a visual 3D-CNN model. Meanwhile, they proposed three types of visual inspection methods: sensitivity analysis, 3D class activation mapping, and 3D-weighted gradient weighted mapping. Some well-known 2D-CNN models were converted into corresponding 3D architectures, and the best accuracy was 79.4%. Kruthika et al. [81] trained an auto-encoder to derive an embedding from the input features of 3D patches. Their proposed model performed the best in all cases, with a validation accuracy of 98.42% for AD and healthy control (HC). Feng et al. [82] stacked LSTM layers on 3D-CNN layers, so that the 3D fully connected CNN layers could obtain deep feature representations and the LSTM could achieve improved performance. Wegmayr et al. [83] used a large number of subjects, and a deep 3D-CNN was researched on a sizeable dataset for the classification task. Ahsan Bin Tufail et al. [84] constructed multiple deep 2D-CNNs for feature learning, and then the whole brain image was passed through two transfer learning architectures. Hosseini-Asl et al. [85] proposed a general feature that can help the neural network model to display AD biomarkers extracted from brain images and classify subjects through fine-tuning methods. The proposed architecture is based on SAE, and the fully connected layer is then fine-tuned. Abrol et al. [86] developed a 3D-CNN based on the ResNet architecture, and experiments were conducted to verify its performance for several binary and multiclass tasks. The model performed better than the SVM and SAE methods. Wang et al. [87] used a 3D dense model to maximize information flow, and each layer of the model was directly connected to all subsequent layers. Then, a probability-based fusion method was exploited to combine the proposed method with different architectures. Cui et al. [88] proposed a longitudinal analysis based on convolution and RNN, and they constructed a 3D-CNN to capture spatial features. Then, three bidirectional gated recurrent units (BGRU) were constructed with cascades at multiple time points on the output of the 3D-CNN to capture timing features. Zhao et al. [89] proposed a 3D multi-information GAN to predict the state of the whole brain, while a 3D-DenseNet-based multi-classification model was also built, which could generate high-quality sMRI on individual 3D brain and multi-information at the baseline time point. 

With the increase in the number of samples, more and more DL methods have been applied to AD diagnosis, and some representative examples in the literature are listed in Table 2.

### 5.2. Parkinson’s Disease

Parkinson′s disease is a chronic neurodegenerative disease that mainly affects the central nervous system and the motor nervous system. The most common early symptoms include tremor, limb stiffness, decreased motor function, and abnormal gait. MDD and anxiety disorders also appear in more than one-third of the cases. Other possible symptoms include perception, sleep, and emotional problems [90]. 

As for ML methods, Solana-Lavalle et al. [91] used seven classifiers, including KNN, SVM, RF, and NB, to classify the MRI images, which act as an assisting tool for diagnosis of PD by using the Parkinson’s Progression Markers Initiative (PPMI) dataset. Filippone et al. [92] applied a multinomial logit classifier to 62 subjects, including 14 subjects with HC, 14 subjects with PD, 16 subjects with progressive supranuclear palsy (PSP), and 18 subjects with multiple system atrophy (MSA). Marquand et al. [93], from the same research group, applied this classifier to a different population with two variations: either healthy controls were included or not in the given classifiers. In addition, the MSA cohort was further divided into Parkinsonian and cerebellar subtypes (MSA-P, MSA-C). 

DL methods have also made a significant contribution. Esmaeilzadeh et al. [94] used an sMRI scan and demographic information of patients to train a 3D-CNN model and found that the upper parietal lobe of the right hemisphere of the brain is essential for the diagnosis of PD. Zhang et al. [95] and McDaniel et al. [96] both used a graph convolutional neural network (GCN) model and presented an end-to-end pipeline, which can directly take brain graphs from multiple views as input and make a prediction on this input. Shinde et al. [97] used a CNN model to create prognostic and diagnostic biomarkers of PD on neuromelanin sensitive magnetic resonance imaging (NMS-MRI). Kollias et al. [98] proposed a model that exploits rich internal descriptions derived by CNN on input data. Furthermore, bidirectional LSTM/gated recurrent units (GRU RNNs) were used to analyze the time course of the input. Moreover, Sivaranjini et al. [99] analyzed T2-weighted MRI scans and performed the classification task through AlexNet. Yasaka et al. [100] applied a CNN model to parameter weighting. They used DWI to calculate a streamline (NOS)-based structural connection group matrix, and applied gradient-weighted category activation mapping (Grad-CAM) to the trained CNN model. The above papers reviewed for the application of AI methods in PD diagnosis are summarized in Table 3. 

### 5.3. Major Depressive Disorder

Major depressive disorder is a mental disorder that is often accompanied by lack of energy, lack of interest in general leisure activities, unexplained pain, and low self-esteem. Patients may have delusions or hallucinations and auditory hallucinations. MDD can adversely affect the daily life, work, education, sleep, eating habits, and overall health of patients [101]. 

The SVM model has made a significant contribution to research on this disease. Jie et al. [102] proposed a feature selection method based on linear SVM and applied it to sMRI and rs-fMRI data. This method can extract distinguishing features from these two kinds of data and classify bipolar disorder (BD) and MDD through the selected features. Rubin-Falcone et al. [103] performed individual-level classification of BD or MDD using gray matter volume (GMV) through SVM, which obtained a combined accuracy of 75%. Deng et al. [104] incorporated diffusion measures of the tracked profiles into SVM and discovered new biomarkers. Jing et al. [105] used SVM to identify every pair of current MDD (cMDD), remitted MDD (rMDD), and HC groups, based on abnormal Hurst exponent features with an AAL-1024 and AAL-90 atlas. Hong et al. [106] exploited SVM to performed a cross-sectional assessment of adolescent/young people, so as to distinguish suicide attempters from those who have suicidal ideas but have not attempted suicide. Hilbert et al. [107] used SVM on generalized anxiety disorder (GAD), MDD, and HC subjects as a case classification and to distinguish GAD from MD as a disorder classification. Guo et al. [108] proposed a new method for generating a high-order minimum spanning tree function to connect the network. In addition, they applied multi-kernel SVM to the selected features to obtain classification results. 

The applications of DL models are less than those of ML, but good results are also achieved. Zeng et al. [109] clustered the voxels within the perigenual cingulate cortex into two subregions, according to their unique functional connection mode in the rest state. It was shown that the unsupervised ML method based on maximum margin clustering can extract enough information from the sub-cingulate functional connection graph to distinguish the depressed patients from the HC. The level of clustering agreement and the individual level of classification agreement both reached 92.5%. Zhao et al. [110] proposed a GAN based on functional network connectivity (FNC). The discriminator and generator of the proposed GAN model both have four fully-connected layers. Jun et al. [111] used spectral GCNs based on a population graph to successfully integrate effective connectivity (EC) and non-imaging phenotypic information. The above papers on the application of AI methods in MDD diagnosis are summarized in Table 4.

### 5.4. Schizophrenia

Schizophrenia, a major psychiatric disorder [112], is a devastating illness that results in chronic impairments in cognition, emotion, and behavior [113,114]. Patients with SCZ usually suffer from other psychological health problems, such as anxiety, clinical depression, or substance abuse disorder [115]. The symptoms of this disease usually appear gradually, and generally begin in early adulthood and last for a long time [116]. The detection of the early symptoms of SCZ through neuroimaging, in a timely and effective manner, is essential for the prevention and diagnosis of SCZ.

In terms of ML models, Jo et al. [117] used network analysis and ML methods to classify SCZ and HC. Bae et al. [118] used an SVM model, and nine features were selected as input. The results suggest that there are significant differences between HC and SCZ subjects regarding the regional brain activity detected with fMRI. Pläschke et al. [119] compared the classification performance of a given network with that of predefined networks of each group and all groups, and SVM was used in this experiment. Ulloa et al. [120] designed a data-driven simulator to generate synthetic samples, and a 10-layer ANN was trained on continuously generated synthetic data, which greatly improved the generalization ability of the model. Kim et al. [121] learned the features from low-level to high-level through an ANN model. In this model, L1-regularization was added to each hidden layer to control the sparsity of weights.

DL models have also been extensively exploited. Kadry et al. [122] used brain MRI slices of T1 modality to detect Schizophrenia, a pre-trained VGG16 model was constructed, and the deep-features extracted were optimized with the slime-mold-algorithm. Pinaya et al. [123] created an SAE model using sMRI data obtained from 1113 healthy people, and they used the model to estimate total and regional neuroanatomical deviation in individual patients, using two independent data sets. Yan et al. [124] proposed a multi-scale RNN model, which enabled direct classification of 558 subjects with SCZ and 542 subjects with HC through time courses of fMRI data. Compared with the existing popular algorithms, such as SVM and RF, SZC achieves a significantly improved classification accuracy. Mahmood et al. [125] proposed a self-supervised pre-training method that can directly pre-train the fMRI dynamics of healthy control subjects and transfer the learning to a smaller SCZ dataset. Patel et al. [126] trained an SAE model on each brain region. The input layer directly uses the time series of voxels, which ensures that the model retains much of the original information. Zeng et al. [127] constructed a multi-site rs-fMRI dataset. Meanwhile, an SAE model with optimized discriminants was used to learn the functional connections of the whole brain. Qureshi et al. [128] established a classification framework based on 3D-CNN, which takes an independent component correlation algorithm (ICA) function network graph as input. The results indicate that these ICA mappings can be used as highly discriminative features to distinguish SCZ. Qi and Tejedor [129] used correlation analysis and an AE model to fuse multi-modal features and obtained better results than the trimming features used in the baseline system. The papers reviewed above on the application of AI methods in SCZ diagnosis are summarized in Table 5.

### 5.5. Attention-Deficit/Hyperactivity Disorder

Attention-deficit/hyperactivity disorder is a mental disease of neurodevelopmental disorders [130,131]. Patients with this disease usually suffer from difficulties in concentrating, being overactive, and doing things without considering the consequences, etc. In addition, patients may exhibit behaviors that are not age-appropriate and have difficulty in emotional regulation or executive function, due to lack of attention [132]. This disease can also be related to other mental disorders or drug abuse [133]. A plethora of studies have been conducted for the diagnosis of ADHD.

ML methods play an important role in the classification task. Luo et al. [134] applied ensemble learning on multimodal neuroimaging data. The bagging-based ensemble learning techniques based on a SVM model achieved the best results. Du et al. [135] exploited graph kernel principal component analysis (PCA) to extract features from the discriminative subnetworks and adopted the SVM model for classification. Iannaccone et al. [136] applied a variety of structural brain patterns to the Flanker/NoGo task and detected error processing and inhibition through functional activation on the sMRI data. Then, they used SVM to make predictions. Eslami and Saeed [137] used a model based on a KNN classifier and also designed a model selection method to select the value of k for KNN. Shao et al. [138] proposed an improved RF method that combines functional connectivity (FC) and low-frequency fluctuation amplitude (ALFF). Moreover, synthetic minority oversampling technology was exploited to generate minority ethnic group cascading feature samples, thus making the distribution of the sample data more balanced. Chen et al. [139] designed a dual-subspace classification algorithm, and SVM was taken as the feature selection strategy for its high computational efficiency. Sen et al. [140] input structural texture and FC features to an SVM classifier and explored a series of three learners.

Extensive research has also been done on DL-based classification tasks. Mao et al. [141] constructed a 4D-CNN model based on 3D-CNN. Since an rs-fMRI image can be regarded as a series of brain 3D models constructed over time, they proposed several spatio-temporal computing methods and fusion models. Yao and Lu [142] proposed an improved GAN model with a data enhancement function, which exploited Wasserstein distance and two-level distance constraints to enhance the data of subjects and control groups. The ability of the classifier was improved by the data generated by the proposed model. Wang et al. [143] introduced a dilated 3D-CNN method and also proposed a framework based on this method. These two methods can classify individual MRI images and image sequences. Atif Riaz et al. [144] proposed a neural network architecture based on a 2D-CNN to diagnose ADHD. The model takes fMRI pre-processed time series signals as input and outputs a diagnosis. The papers reviewed above on the application of AI methods in ADHD diagnosis are summarized in Table 6.

### 5.6. Autism Spectrum Disorder

Autism spectrum disorder is a disease caused by brain developmental disorders. It features emotional, verbal, and nonverbal expression difficulties, and social interaction disorders. Patients with this disease display restrictive behaviors and repetitive actions [145]. These symptoms become serious gradually, but some children with autism have a normal or near-normal early stage of development before one or more idiosyncratic features of autism appear, such as language regression. Therefore, the specific period of early development is less affected by autism, which makes diagnosis difficult [146]. With the development of AI technology, more and more auxiliary diagnostic methods have been proposed.

As for the application of ML models, Chen et al. [147] used an SVM model with features of whole brain FC-networks, which were constructed in specific frequency bands. The study indicates that frequency specified FC-networks have the potential to become biomarkers for ASD. Chen et al. [148] exploited intrinsic functional connectivity between a set of functionally defined ROIs (region of interest) for ML-based diagnostic classification. This approach obtained a higher accuracy than SVM. Plitt et al. [149] performed leave-one-out cross-validation on a dataset, considering nine ML models. LR and SVM performed the best, with an average accuracy of 73.33% and 73.89% for distinguishing ASD from typical development (TD). Wang et al. [150] proposed a multi-site adaptation framework with low-rank representation and then applied a SVM/KNN classifier on the target data. The proposed method exhibited better effectiveness than that of several state-of-the-art methods. Eslami and Saeed [151] fed the features extracted by MLP into an SVM classifier to investigate the discriminative power of these features. A technique called auto-tune models (ATM) was exploited to optimize the hyperparameters of the SVM model. 

DL models have also been applied to various applications. El-Gazzar et al. [152] exploited local and global spatio-temporal structures through 3D-CNN and 3-D Convolutional LSTM, which provided an alternative method for hard-coded features and summary measures, to reduce the dimensionality. Li et al. [153] trained an SAE model and then used transfer learning for ASD classification. Yao and Lu [142] proposed a GAN model to augment brain functional data. The experimental results indicated that the classification accuracy was greatly improved by data augmentation. Kong et al. [154] constructed an individual brain network for each subject and extracted 3000 top connectivity features between each pair of ROIs, which were later used to perform ASD/TC classification via an SAE classifier. Hazlett et al. [155] implemented an SAE-based model that exploited the surface area information of infant brain MRI images to predict the 24-month autism diagnosis of high-family-risk children. Khosla et al. [156] took the functional connectivity of each voxel with the target ROI as input features, and they exploited a 3D-CNN framework for classification. Ktena et al. [157] implemented a GCN model which considered the graph structure in terms of the similarity between two graphs. Anirudh and Thiagarajan [158] proposed another version of the GCN model to reduce the sensitivity of the model in the initial graph construction step. Yao et al. [159] used a multi-scale triplet GCN to overcome the spatial limitation of a single template. Dvornek et al. [160] directly input rs-fMRI time series data, instead of the pre-calculated measures of connective tissue brain function, to an LSTM model. The papers reviewed above on the application of AI methods in ASD diagnosis are summarized in Table 7.

## 6. Conclusions and Discussion

Six traditional ML algorithms and seven DL algorithms were reviewed in this paper. Then, six typical neurological and psychiatric diseases were discussed, and the recent representative research works exploiting ML and DL methods to diagnose these diseases were summarized. The use of artificial intelligence to assist in the diagnosis of disease will be a crucial method in the near future.

In the early days, ML was widely used because of its simplicity and efficiency. Reducing the dimensionality of features and selecting the most important features are crucial parts of ML. Based on a series of feature selection algorithms, different types of subjects are classified through a classifier. Although deep learning does not need feature selection, DL-based MRI image classification often requires various methods of pre-processing of images, thus failing to achieve real end-to-end learning. Furthermore, because the sample size of the dataset is not big enough, the deep learning models are easy to overfit, and the generalization ability of the models is not strong enough. Moreover, DL depends highly on the configuration of hyper-parameters, which may make its performance fluctuate dramatically, and sometimes experience is a big factor affecting the result.

Compared with traditional ML methods, DL does not show advantages in classification. In terms of overhead, DL consumes more resources than traditional ML. Recently, with the continuous increase of the amount of MRI data and the continuous improvement of DL methods, more and more DL methods have been applied to MRI image classification tasks. However, the growth of the data set still cannot meet the requirements of DL models. In this case, traditional ML is still a popular and important technology for medical diagnosis.

Among the six traditional ML models, SVM is most used, because of its advantages of computational overheads and generalization performance. Meanwhile, different imaging methods usually reflect different temporal and spatial scale information of the brain. For example, sMRI data reflects the spatial structure information of the brain, while fMRI data provides information on the time domain. Among all the DL models, 3D-CNN aggregates the spatial structure features of the image very well, based on the data characteristics of sMRI. Since timing features can be extracted by RNN, transfer learning can transfer the knowledge or patterns learned in a certain field to different but related fields. This improves the generalization ability of the training model on a small sample to a certain extent.

The combination of traditional ML and DL models has achieved unexpected results. Researchers have exploited DL models to automatically extract features. The combination of these features with traditional ML feature selection and classification algorithm models contributed to better results than that of a single model. In addition, the doctor′s clinical diagnosis plays an important role in the classification model, and currently the main objective is for AI to assist in diagnosis.

To date, there is no real ML or DL model designed for human brain MRI data, because most models are migrated from other fields. For example, the 3D-CNN was migrated from the 2D-CNN on two-dimensional images. The exponential increase of computing resource consumption brought about by the migration process poses great challenges for clinical applications. Meanwhile, different diseases, or even the same disease on different data sets, different models, or different parameters of the same model, can lead to different results. This makes the models difficult to interpret. Furthermore, the prediction accuracy achieved by DL-based methods is far from sufficient for clinical diagnosis. Thus, there is still a long way to go for DL-based methods to be used as auxiliary diagnosis methods. Moreover, artificial intelligence in medical diagnosis is still a relatively new method, and many clinicians still do not believe in its reliability and sensitivity. Thus, it is critical to integrate it into clinical practice without harming clinical expertise.

This review paper is based on the author′s own analysis and summary of the literature, and although we tried to remain objective in the analysis process, it is still highly subjective and all findings are based on personal opinions. This review paper only covers part of the scientific research results of the past ten years. The sources of article retrieval were Pubmed and Web of Science, so readers need to understand the limitations of this review paper in terms of time and sources.

## Figures and Tables

**Table 1:** Review papers of the application of ML and DL methods to MRI data.

Reference	Year	Number of Papers Reviewed	Years Covered	Pathology/Anatomical Area
[10]	2020	42	2015–2019	Alzheimer’s disease, Parkinson’s disease, Schizophrenia
[22]	2020	155	2010–2019	Neurological disorders, Alzheimer’s disease, Schizophrenia, Brain tumor, Cerebral artery, Parkinson’s disease, Autism spectrum disorder, Epilepsy, Other
[23]	2020	100	2016–2019	Alzheimer’s disease, Parkinson’s disease, Autism spectrum disorder, Schizophrenia
[24]	2019	56	2016–2018	Brain age, Alzheimer’s disease, Vascular lesions, Brain extraction, etc.
[25]	2020	22	2010–2019	Schizophrenia
[26]	2017	300	1995–2017	Image/exam classification, Object or lesion classification, Object or lesion detection, Object or lesion detection, Lesion segmentation, etc.
[27]	2019	65	2008–2018	Schizophrenia, Autism spectrum disorder, Parkinson’s disease, Depression, Substance Abuse disorder, Epilepsy, etc.
[7]	2017	85	-	Model/Algorithm, Alzheimer’s disease, etc.

**Table 2:** Literature review of AD diagnosis using ML and DL methods.

Reference	Model	Year	Modality	Subjects	Training Set/Test Set	Accuracy (%)
[75]	NB, ANN, KNN, SVM	2020	T1-w	78 AD, 72 HC	373/150	98 for hybrid modeling
[76]	SVM	2015	T1-w, DTI, rs-fMRI	28 AD, 25 HC	leave-one-out	rs-fMRI-74, DTI-85, GM volume-81
[77]	SVM, RF	2014	T1-w	200 AD, 231 HC, 164 pMCI, 100 sMCI, 130 uMCI	10-fold cross-validated	pMCI/sMCI-81.72
[78]	SVM, SRC	2012	T1-w	198 AD, 225 MCI, 229 HC	-	AD/HC-90.8, MCI/HC-87.85
[79]	2D-CNN, Transfer learning	2021	rs-fMRI	25 HC, 13 MCI, 25 EMCI, 25 LMCI, 25 SMC, 25 AD	51443/27310	EMCI/LMCI-99.45, AD/HC-75.12, HC/EMCI-96.51, HC/LMCI-74.91, EMCI/AD-99.90, LMCI/AD-99.34, MCI/EMCI-99.98
[80]	3D-CNN	2018	T1-w	47 AD, 56 HC	103/8	79.4 ± 0.070
[81]	AE, 3D-CNN	2019	T1-w, PET	345 AD, 991 MCI, 605 NC	3/1	MCI/AD-94.6, NC/AD-92.98, NC/MCI-94.04
[82]	RNN, 3D-CNN	2019	T1-w	93 AD, 76 pMCI, 128 sMCI, 100 HC	10-fold cross-validation	AD/HC-94.82, pMCI/HC-86.36, sMCI/HC-65.35
[83]	3D-CNN	2018	T1-w	6218 HC, 8268 MCI, 4076 AD	-	AD/HC-86, MCI/AD-72, MCI/HC-67, MCI/AD/HC-60.2
[84]	2D-CNN, Transfer learning	2020	T1-w	90 AD, 90 HC	9-fold Cross-Validation, etc.	99.45
[85]	AE, 3D-CNN	2016	T1-w	70 AD, 70 MCI, 70 NC	10-fold Cross-Validation	AD/MCI/HC-94.8, AD+MCI/HC-95.7, AD/HC-99.3, AD/MCI-100, MCI/HC-94.2
[86]	3D-CNN	2020	T1-w	157 AD, 189 pMCI, 245 sMCI, 237 HC	5-fold Cross-Validation	AD/HC-89.3, pMCI/HC-86.5, sMCI/AD-87.5, sMCI/pMCI-75.1
[87]	3D-CNN	2019	T1-w	221 AD, 297 MCI, 315 HC	10-fold Cross-Validation	MCI/AD-93.61, MCI/HC-98.42, AD/HC-98.83, AD/HC/MCI-97.52
[88]	3D-CNN, RNN	2019	T1-w	198 AD, 167 pMCI, 236 sMCI, 229 HC	5-fold Cross-Validation	AD/HC-91.33, pMCI/sMCI-71.71
[89]	3D-CNN, GAN	2020	T1-w	151 AD, 341 MCI, 113 HC	7-fold Cross-Validation	MCI/AD/HC-76.67, pMCI/sMCI-78.45

**Table 3:** Literature review of PD diagnosis using ML and DL methods.

Reference	Model	Year	Modality	Subjects	Training Set/Test Set	Accuracy (%)
[91]	KNN, SVM, RF, NB et al.	2021	T1-w	226 male PD, 86 male HC, 104 female PD, 64 female HC	10-fold Cross-Validation	Male-99.01, Female-96.97
[92]	LR, SVM	2013	T1-w, T2-w, DTI	14 HC, 14 PD, 16 PSP, 18 MSA	4-fold Cross-Validation	62.7
[93]	LR, SVM	2013	T1-w, T2-w, DTI	17 PSP 19 MSA, 14 IPD, 19 HC	leave-one-out	PSP/IPD/MSA-91.7, PSP/IPD/HC/MSA-73.6 ,PSP/IPD/MSA-P/MSA-C-84.5 PSP/IPD/HC/MSA-P/MSA-C-66.2
[94]	3D-CNN	2018	T1-w	292 male PD, 134 male HC, 160 female PD, 70 female HC	17:1	100
[95]	GNN	2018	T1w, DTI	596 PD, 158 HC	5-fold Cross-Validation	-
[96]	GNN	2019	T1w, DTI	117 PD, 30 HC	-	92.14
[97]	2D-CNN	2019	T1-w, T2-w, DWI	45 PD, 20 APS, 35 HC	5-fold Cross-Validation	80
[98]	2D-CNN, RNN	2017	T1-w	55 PD, 23 Parkinson-related syndromes	78/26	94
[99]	2D-CNN, Transfer learning	2020	T2-w	100 PD, 82 HC	8/2	88.9
[100]	2D-CNN	2021	T1-w, DWI	115 PD, 115 HC	5-fold Cross-Validation	81

**Table 4:** Literature review of MDD diagnosis using ML and DL methods.

Reference	Model	Year	Modality	Subjects	Training Set/Test Set	Accuracy (%)
[102]	SVM	2015	rs-fMRI	21 BD, 25 MDD	leave-one-out	92.1
[103]	SVM	2018	T1-w	26 BD, 26 MDD	leave-two-out	75
[104]	SVM	2018	DTI	31 BD, 36 MDD	-	68.3
[105]	SVM	2017	rs-fMRI	19 cMDD, 19rMDD, 19 HC	leave-one-out	cMDD/HC-87 rMDD/HC-84 cMDD/rMDD-89
[106]	SVM	2021	T1-w	66 MDD	leave-one-out	78.59
[107]	SVM	2017	T1-w	19 GAD, 14 MDD, 24 HC	leave-one-out	GAD+MDD/HC -90.10, GAD/MDD-67.46
[108]	SVM	2017	rs-fMRI	38 MDD, 28 HC	-	97.54
[109]	SVM-based	2014	rs-fMRI	24 MDD, 29 HC	leave-one-out	92.5
[110]	GAN	2020	rs-fMRI	269 MDD, 286 HC	10-fold Cross-Validation	80.7
[111]	GNN	2020	rs-fMRI	29 MDD, 44 HC	10-fold Cross-Validation	74.1

**Table 5:** Literature review of SCZ diagnosis using ML and DL methods.

Reference	Model	Year	Modality	Subjects	Training Set/Test Set	Accuracy (%)
[117]	SVM, RF, NB	2020	T1-w	48 SCZ, 24 HC	10-fold Cross-Validation	68.6
[118]	SVM	2018	fMRI	21 SCZ, 54 HC	10-fold Cross-Validation	92.1
[119]	SVM	2017	rs-fMRI	86 SCZ, 84 HC	10-fold Cross-Validation	72
[120]	ANN	2015	T1-w	198 SCZ, 191 HC	10-fold Cross-Validation	-
[121]	ANN	2015	rs-fMRI	50 SCZ, 50 HC	5-fold Cross-Validation	85.8
[122]	2D-CNN	2021	T1-w	500 HC slices, 500 SCZ slices	-	94.33
[123]	SAE	2019	T1-w	35 SCZ, 40 HC	10-fold Cross-Validation	-
[124]	RNN	2019	fMRI	558 SCZ, 542 HC	5-fold Cross-Validation	83
[125]	Transfer learning	2019	rs-fMRI	151 SCZ, 160 HC	8/1	-
[126]	SAE	2016	fMRI	72 SCZ, 74 HC	10-fold Cross-Validation	92
[127]	SAE	2018	rs-fMRI	357 SCZ, 377 HC	5-fold Cross-Validation	85
[128]	3D-CNN	2019	rs-fMRI	72 SCZ, 74 HC	10-fold Cross-Validation	98.09
[129]	AE	2016	T1-w, fMRI	69 SCZ, 75 HC	-	-

**Table 6:** Literature review of ADHD diagnosis using ML and DL methods.

Reference	Model	Year	Modality	Subjects	Training Set/Test Set	Accuracy (%)
[134]	SVM, KNN, LR, NV, RF et al.	2020	T1-w, DTI, fMRI	36 ADHD, 36 HC	5-fold Cross-Validation	ADHD/HC-81.6, ADHD-P/ADHD-R-78.3
[135]	SVM	2016	rs-fMRI	118 ADHD, 98 HC	9/1	94.91
[136]	SVM	2015	T1-w, fMRI	18 ADHD, 18 HC	leave-one-out	77.78
[137]	KNN	2018	fMRI	973	-	81
[138]	RF	2019	fMRI	78 ADHD, 116 HC	541/128	82.73
[139]	SVM	2020	rs-fMRI	272 ADHD, 361 HC	leave-one-out	88.1
[140]	SVM	2018	T1-w, fMRI	279 ADHD, 279 HC	558/171	68.9
[141]	3D-CNN-based	2019	rs-fMRI	359 ADHD, 429 HC	626/126	71.3
[142]	GAN	2019	fMRI	487	-	90.2
[143]	3D-CNN	2019	T1-w	587	5-fold Cross-Validation	76.6
[144]	2D-CNN	2020	rs-fMRI	359	349/117	73.1

**Table 7:** Literature review of ASD diagnosis using ML and DL methods.

Reference	Model	Year	Modality	Subjects	Training Set/Test Set	Accuracy (%)
[147]	SVM	2016	fMRI	112 ASD, 128 HC	leave-one-out	79.17
[148]	RF	2015	fMRI	126 ASD, 126 TD	137/43	91
[149]	LR, SVM et al.	2014	rs-fMRI	148 ASD, 148 TD	leave-one-out	73.89
[150]	SVM, KNN	2020	rs-fMRI	250 ASD, 218 HC	Cross-Validation	73.44
[151]	SVM	2019	fMRI	187 ASD, 183 HC	5-fold Cross-Validation	80
[152]	3D-CNN, RNN	2020	fMRI	184 ASD, 110 TD	5-fold Cross-Validation	77
[153]	SAE, Transfer learning	2018	rs-fMRI	149 ASD, 161 HC	Cross-Validation	70.4
[142]	GAN	2019	fMRI	454	-	87.9
[154]	SAE	2019	T1-w	78 ASD, 104 TD	10-fold Cross-Validation	90.39
[155]	SAE	2017	T1-w	34 ASD, 145 HC	10-fold Cross-Validation	81
[156]	3D-CNN	2018	rs-fMRI	379 ASD, 395 HC	-	73.3
[157]	GNN	2017	fMRI	403 ASD, 468 HC	5-fold Cross-Validation	-
[158]	GNN	2019	rs-fMRI	872	-	70.86
[159]	GNN	2021	fMRI	1160	-	86
[160]	RNN	2017	fMRI	539 ASD, 573 TD	Cross-Validation	68.5
